# High Sensitivity of Ammonia Sensor through 2D Black Phosphorus/Polyaniline Nanocomposite

**DOI:** 10.3390/nano11113026

**Published:** 2021-11-11

**Authors:** Zuquan Wu, Lei Liang, Shibu Zhu, Yifan Guo, Yao Yao, Yong Yang, Shifu Gu, Zuowan Zhou

**Affiliations:** 1 School of Electrical Engineering and Electronic Information, Xihua University, Chengdu 611743, China; zuquanwu@aliyun.com (Z.W.); liangleiaaa@aliyun.com (L.L.); dr_yongyang@163.com (Y.Y.); gushifu@139.com (S.G.); 2 Key Laboratory of Advanced Technologies of Materials (Ministry of Education), School of Materials Science and Engineering, Southwest Jiaotong University, Chengdu 610031, China; yfguo@my.swjtu.edu.cn; 3 Xi’an Aerospace Composites Research Institute, Xi’an 710025, China; 4 School of Automation Engineering, University of Electronic Science and Technology of China, Chengdu 610054, China; yaoyao386@yahoo.com

**Keywords:** black phosphorus/PANI nanocomposite, thin film, gas sensor, ammonia

## Abstract

Recently, as a two-dimensional (2D) material, black phosphorous (BP) has attracted more and more attention. However, few efforts have been made to investigate the BP/polyaniline (PANI) nanocomposite for ammonia (NH_3_) gas sensors. In this work, the BP/PANI nanocomposite as a novel sensing material for NH_3_ detection, has been synthesized via in situ chemical oxidative polymerization, which is then fabricated onto the interdigitated transducer (IDTs). The electrical properties of the BP/PANI thin film are studied in a large detection range from 1 to 4000 ppm, such as conduction mechanism, response, reproducibility, and selectivity. The experimental result indicates that the BP/PANI sensor shows higher sensitivity and larger detection range than that of PANI. The BP added into PANI, that may enlarge the specific surface area, obtain the special trough structure for gas channels, and form the *p–π* conjugation system and *p–p* isotype heterojunctions, which are beneficial to increase the response of BP/PANI to NH_3_ sensing. Meanwhile, in order to support the discussion result, the structure and morphology of the BP/PANI are respectively measured by Fourier transform infrared spectroscopy (FTIR), ultraviolet-visible spectroscopy (UV−vis), transmission electron microscopy (TEM), and field emissions scanning electron microscopy (SEM). Moreover, the sensor shows good reproducibility, and fast response and recovery behavior, on NH_3_ sensing. In addition, this route may offer the advantages of an NH_3_ sensor, which are of simple structure, low cost, easy to assemble, and operate at room temperature.

## 1. Introduction

The environment consists of various gases, such as nitrogen (N_2_), oxygen (O_2_), carbonic oxide (CO), carbon dioxide (CO_2_), nitrogen dioxide (NO_2_), and ammonia (NH_3_). Among these gases, NH_3_ is beneficial to human beings, which is widely used in various applications, for instance, refrigeration, refining, manufacturing, cleaning, and nitrogenous fertilizers [1,2]. Meanwhile, NH_3_ is a toxic and dangerous gas which is flammable at concentrations of ca. 15–28% by volume in air [3]. Therefore, it is considered as a high-hazard, toxic, industrial chemical that can be used as a crude terrorist weapon [1]. In addition, for human beings, the skin, eyes, and respiratory tract may be injured at high concentrations of NH_3_ (ca. >300 ppm). Due to the huge demand of the NH_3_ sensor market, all kinds of sensitive materials have been reported as NH_3_ sensors, such as inorganic [4], metal oxides [5], carbonaceous material [6,7], conducting polymers [8], composite material [9,10], and so on. However, a few disadvantages of gas sensors have been gradually found, such as their being expensive, having chemical instability, and their high temperature operation, high power consumption, and high cost maintenance [9,11]. Hence, it is a beneficial and significant job for us to focus on developing high-performance NH_3_ sensors, which are low cost, reliable, nontoxic, and able to operate at room temperature in practice.

As a famous conducting polymer, polyaniline (PANI) is frequently used for fabricating humidity [11] and gas [12] sensors owing to its facile synthesis, environmental stability, high sensitivity, fast response, low cost, reversible redox reaction, and room temperature operation [9,13]. Over the past few decades, there has been an effective route for enhancing the mechanical strength and characteristics of gas sensors via combination of the conducting polymers and inorganic counterparts to form composites [9,14]. Therefore, a variety of inorganic nanofillers have been added to PANI to form composites which are used for gases detection, such as PANI/In_2_O_3_ for H_2_, NO_2,_ or CO gases [15], PANI/TiO_2_ for NH_3_ or CO gases [9], multiwall carbon nanotube (CNT)/PANI or graphene/PANI [13] for hydrogen gas [14], functionalized single-walled CNT/PANI for NH_3_, NO_2_, or H_2_S gases [16], and WO_3_ hollow spheres@ PANI for NH_3_ gas [17]. 

In recent years, as a two-dimensional (2D) material, black phosphorous (BP) has been rediscovered, and has also attracted extensive attention from the academic community [18]. BP is one of the allotropes of phosphorus that possess physicochemical characteristics due to its honeycomb sheet with unique trough-like structures [19]. In general, BP has been widely used for batteries, water splitting, photovoltaic solar cells, photodetectors, and sensors [20], such as field effect transistors [21], nitrogen dioxide sensors [22], humidity sensor [18], methanol vapor sensor [23], biosensor [24], etc. Unfortunately, BP has extremely limited application due to the lack of long-term stability, and because it is easily oxidized and degraded upon exposure to ambient conditions [20,25]. In order to avoid rapid degradation, inert aluminum oxide or boron nitride as encapsulating layer has been deposited on the BP surface [20,21]. The combination of BP with other materials has also solved the problem and obtained the novel composites. Wu et al. [26] developed a BP/CNT hetero-structured material, which was assembled into non-woven fabrics toward achieving a high energy density flexible supercapacitor. Cai et al. [27] synthesized porous graphene functionalized BP composite material and used it in a bisphenol sensor. Sajedi-Moghaddam et al. [28] reported a BP/PANI hybrid material and studied the performance of a capacitor. Durai et al. [29] prepared PANI sheathed BP via the electrochemical polymerization, which was evaluated for electrochemical detection of ascorbic acid and hydrazine. Qian et al. [25] reported a 4-azidobenzoic acid modified BP, which was able to dye molecules and form a composite film, for acid and alkali gas detection. Wang et al. [30] reported BP decorated with titanium dioxide and used it as a sensing layer for 0.5–30 ppm NH_3_ detection.

As mentioned, utilizing BP to modify PANI, an enhanced response of gas sensors based on a BP/PANI hybrid composite may be realized. Until now, to the best of our knowledge, few efforts have been made to investigate BP/PANI nanocomposites for NH_3_ gas sensors. In this work, BP/PANI nanocomposite as a novel sensitive material for NH_3_ gas detection has been synthesized by in situ chemical oxidative polymerization, and is then fabricated onto an interdigitated transducer (IDTs) using the drop-coating method. In the meantime, in order to support discussion results, the structure and morphology of BP/PANI is characterized by Fourier transform infrared spectroscopy (FTIR), ultraviolet-visible spectroscopy (UV−vis), transmission electron microscopy (TEM), and field emissions scanning electron microscopy (SEM), respectively. The electrical properties of the BP/PANI thin film are studied in a large detection range from 1 to 4000 ppm, and include the conduction mechanism, response, reproducibility, and selectivity. The experimental results indicate that the BP/PANI thin film sensor shows a higher sensitivity and larger detection range than that of PANI. The sensitivity and detection range of BP/PANI are notably enhanced because BP plays an important role in gas diffusion. The BP added to PANI, that may enlarge the specific surface area, obtain the special trough structure for gas channels, and form the *p-π* conjugation system and *p*-*p* heterojunctions, which are beneficial to increase the response of BP/PANI to NH_3_ sensing. Moreover, the sensor shows good reproducibility and a fast response and recovery behavior for NH_3_ sensing. Further research exploring the stability of the BP/PANI nanocomposite is summarized in our next study.

## 2. Materials and Methods

### 2.1. Materials

Aniline (Ani) was treated by reduced pressure distillation and stored below 0 °C before use. BP was obtained from Nanjing XFNANO Materials Tech Co., Ltd. (Nanjing, China). Ani, ammonium peroxydisulfate (APS; (NH_4_)_2_S_2_O_8_), hydrochloric acid (HCl), and ethanol were purchased from Chengdu Kelong Chemical Reagent Co., Ltd. (Chengdu, China). The standard NH_3_ (10,000 ppm) and pure N_2_ gases were obtained from NIMTT (Chengdu, China). All of the chemical reagents were analytical purity and used without any further purification. Deionized water (18.2 MΩ∙cm^−1^ resistivity, Milli-Q) was used in the experiments.

### 2.2. Synthesis of BP/PANI Nanocomposite

The BP/PANI was doped with HCl, which was synthesized by in situ polymerization of the Ani monomer in the presence of BP aqueous solution, with APS as the oxidant. It was a typical operation using a similar procedure to that of our previous work [31]. First, 2 mmol Ani, 4 mg BP, and 1 M HCl were, respectively, mixed in 80 mL deionized water. In the whole process, an ice bath (0–5 °C) was used to cool the solution, which was magnetically stirred for 20 min to form BP/Ani micelles. Successively, 2 mmol APS was dissolved in 20 mL deionized water and added to the above cooled solutions. Then, the polymerization reaction was carried out under moderate stirring for 16 h in an ice bath. The resulting dark green precipitates were filtered and washed with deionized water and ethanol, respectively, several times, followed by drying overnight under vacuum in an oven at 25 °C. For comparison, PANI was synthesized using the same process as BP/PANI but without BP. 

### 2.3. Device Fabrications and Apparatus

**Figure 1:**
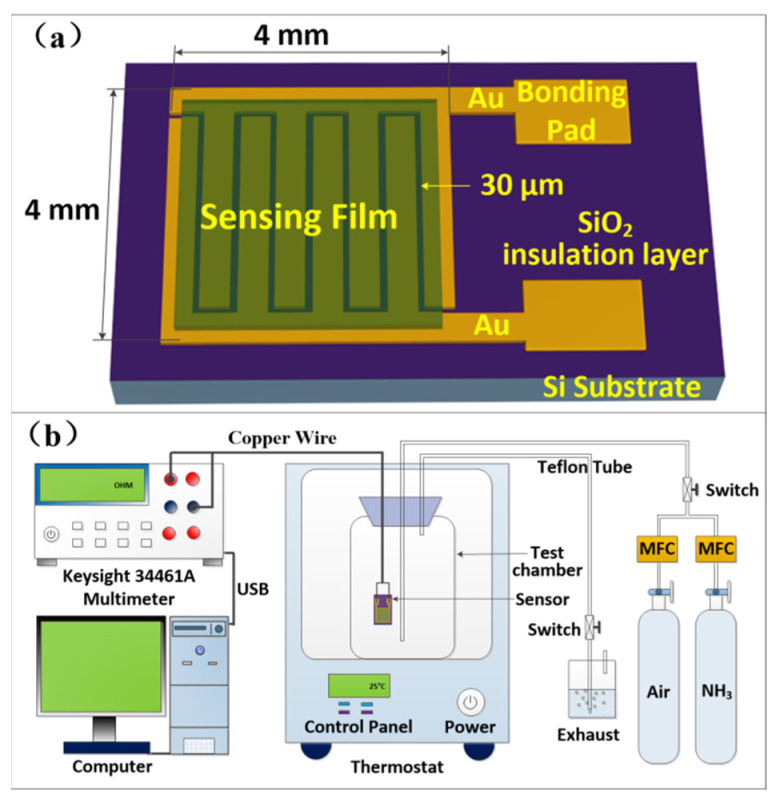
Schematic illustration of (**a**) interdigitated transducers (IDTs) with sensing film and (**b**) the NH_3_ response test apparatus.

An n-type doped Si (100) substrate was used to fabricate the IDTs transducers, and was carried out using a similar procedure to our earlier work [32]. The IDTs were respectively used to prepare the NH_3_ sensors, based on BP/PANI and PANI thin films, by the drop-coating method, as shown in Figure 1a.

Figure 1b shows a schematic diagram of the experimental apparatus for the sensor test. All of the sensors were stored in a sealed box before the experiments and all of the experiments were carried out in a thermostat chamber and controlled at ~25 °C. The pure N_2_ gas was used to purge the airtight test chamber (1 L) about 30 min before the experiment. The concentration of NH_3_ in the test chamber was controlled by mass flow controller (MFC). The resistance changes in the thin films were measured using a digital multimeter (Keysight 34470A, Santa Rosa, CA, USA) and was recorded on a computer, via a USB cable, for data processing.

The morphology of the BP/PANI and PANI nanostructures were respectively analyzed by an INSPECT F50 (FEI, Waltham, MA, USA) SEM and a JEM 1200EX (Jeol Ltd., Tokyo, Japan) TEM. Furthermore, the FTIR spectra, with a resolution of 4 cm^−1^, were analyzed by a TENSOR II spectrometer (BRUKER, Karlsruhebrook, Germany), employing KBr pressed disk. The UV−vis spectra, in the range of 190–900 nm, were analyzed using a UV-2600 spectrometer (SHIMADZU, Kyoto, Japan). 

## 3. Results and Discussion

### 3.1. Characterization: FT-IR, UV−vis, SEM, and TEM of PANI and BP/PANI

**Figure 2:**
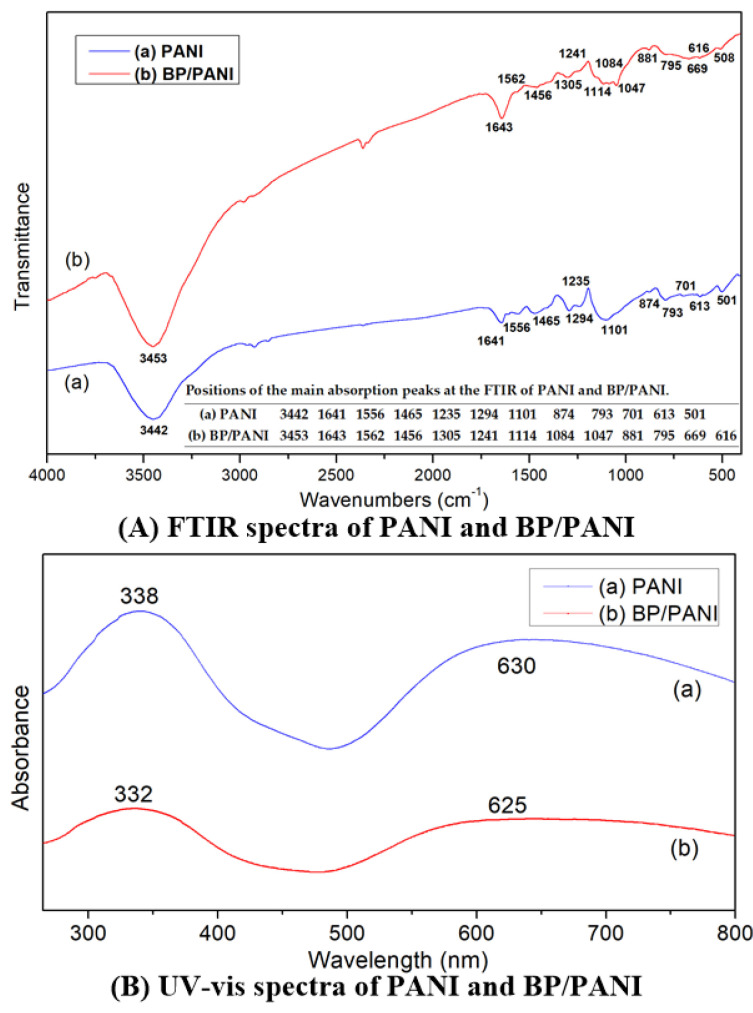
(**A**) FT−IR and (**B**) UV−vis spectra of (a) PANI and (b) BP/PANI.

As shown in Figure 2, the FTIR and UV−vis spectra of PANI and BP/PANI were analyzed, respectively. Figure 2A shows that the FTIR spectra of PANI and BP/PANI were in the range of 400–4000 cm^−1^. As shown in Figure 2A(a), the main characteristic bands of PANI were consistent with the reported references [9,31,32,33,34]. The peak at 3442 was assigned to N–H stretching vibration. The peaks at 1556 cm^−1^ and 1465 cm^−1^ were, respectively, attributed to C=N and C=C stretching mode of quinonoid and benzenoid rings. The bands at 1294 and 1235 cm^−1^ are related to the C–N stretching mode of the benzenoid unit. The peaks at 1101 cm^−1^ and 874 cm^−1^ were assigned to the vibration of C–H. As shown in Figure 2A(b), the FTIR spectra of BP/PANI were almost consistent with PANI and indicated major structural rearrangement. However, it was found that all bands of BP/PANI showed a slight shift in comparison with the pure PANI, such as 1562 cm^−1^, 1456 cm^−1^, 1305 cm^−1^, 1241 cm^−1^, and 1131 cm^−1^. The shift means that there was some bonding interaction between the BP and PANI within the BP/PANI nanocomposite through the C⋯P hybrid.

Figure 2B displays the UV−vis absorption spectra of PANI and BP/PANI. Figure 2B(a) gives the absorption peaks of PANI at 630 nm and 338 nm, which are, respectively, related to the benzenoid-to-quinoid and the π–π* transitions of the benzenoid ring excitonic transition. At the same time, the absorption peaks of BP/PANI are almost identical to that of pure PANI. It can be seen that there is a blue shift of 6 nm in the π–π* transition for the peak of BP/PANI at 332 nm compared to that of PANI. Therefore, BP added into PANI may enhance the intermolecular interaction and extend the energy band gap of BP/PANI [35].

**Figure 3:**
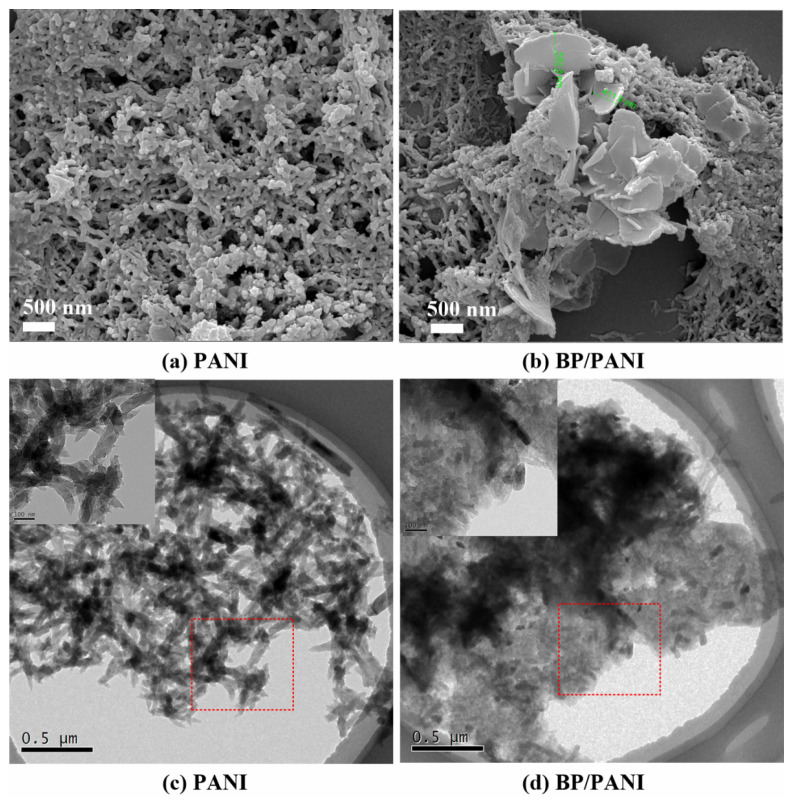
SEM and TEM images of (**a**,**c**) PANI and (**b**,**d**) BP/PANI.

The SEM and TEM images of PANI and BP/PANI nanocomposite thin films are shown in Figure 3. It can be seen that the fibers of PANI and BP/PANI are a meshed structure and all of the PANI dimensions are in the nanometer range. At the same time, it is clearly seen that there are a lot of pores throughout the surface of the thin films, which may benefit the reversibility and response of the gas sensor. Figure 3a shows that the meshed PANI has some pores. Figure 3b shows that PANI also has a lot of pores and is uniformly distributed on the BP (ca. 411–582 nm diameter) surface. There are larger holes in the BP/PANI than there are in pure PANI, because BP plays a key role as support material to form the BP/PANI nanocomposite during chemical polymerization. As shown in Figure 3c,d, the TEM images of PANI and BP/PANI are consistent with those of SEM analysis. Therefore, BP added to PANI to form the BP/PANI nanocomposite may increase the total specific surface area, which may benefit the fast response of the gas sensor.

### 3.2. Gas Response of Sensors

**Figure 4:**
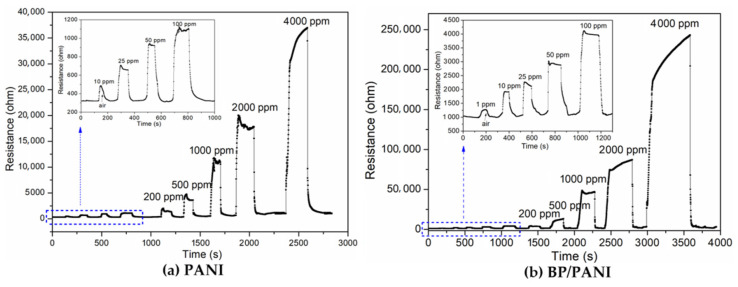
Response transients of (**a**) PANI and (**b**) BP/PANI thin films exposed to different concentrations of NH_3_ at room temperature.

As mentioned in Figure 1b, a thermostat is used to control the temperature of the sensor test process. The initial resistance of PANI (ca. 321 Ω) and BP/PANI (ca. 1011 Ω) sensors was determined in air at room temperature. As shown in Figure 4a,b, the total initial resistance of BP/PANI thin film is clearly higher than that of PANI. The reason for that is not completely clear; thus, probable reasons can be listed as follows. Firstly, the initial resistance of BP (ca. 200 kΩ [30]) is higher than that of PANI, which may increase the total resistance of BP/PANI. Secondly, as a nanofiller, BP is uniformly dispersed in PANI, resulting in chain disorder, deformation, and band structure change of PANI within BP/PANI [36]. Chain disorder and deformation may reduce the degree of delocalization, which leads to π electron hopping transport between the localized states of BP/PANI. Thirdly, the initial resistance of BP/PANI is also influenced by the conjugation length of the PANI chains [9]. In the synthesis process, the concentrations of aniline monomers or nucleation sites are decreased due to the BP added to the solution, which may reduce the chain length and diameter of PANI nanofibers within BP/PANI [37]. Therefore, the high resistance of BP added to PANI, reducing the conjugation length and the chain deformation, may enhance the initial total resistance of BP/PANI. This idea is also supported by FTIR and UV−vis, as analyzed in our previous blueshifts of BP/PANI.

As shown in Figure 4a,b, the response transients of PANI and BP/PANI sensors, exposed to various concentrations of NH_3_ at room temperature. Meanwhile, the inset curves of PANI and BP/PANI reveal the response situation, in a range up to 100 ppm. It can be seen that the resistance of the sensors increases, which are exposed to various concentrations in a large detection range, from 1 to 4000 ppm, and then decrease to the original value, in air. The BP/PANI sensor can detect 1 ppm of NH_3_ gas, which is lower than that of PANI (10 ppm); however, the NH_3_ sensing mechanism of the BP/PANI thin films is not fully understood at the present. The probable explanation can be mainly given as follows; on the one hand, the following equation,

(1) $$\mathrm{BP}/\mathrm{PANI}-H^{+}+{\mathrm{NH}}_{3}⇆\mathrm{BP}/\mathrm{PANI}+{\mathrm{NH}}_{4}^{+}$$

is a reversible reaction in the sensitive BP/PANI thin film for NH_3_ [38]. It can be seen that the protons (H^+^) transfer from the –NH– groups of PANI to the NH_3_ molecules and form ammonium ions (${\mathrm{NH}}_{4}^{+}$), resulting in the emeraldine PANI turning into its base form [39] and an overall density reduction of majority carriers within BP/PANI [30]. That is to say, the NH_3_ molecules decrease the BP/PANI doping level, which may increase the total resistance of BP/PANI [40]. On the other hand, BP/PANI thin films produce swelling and deformation owing to the absorption of the NH_3_ molecules into the nanocomposite thin film, which may reduce the degree of connectivity of the thin film. It seems that the swelling and deformation of the thin film dramatically increases the total resistance of BP/PANI when it is exposed to NH_3_ [12,41].

**Figure 5:**
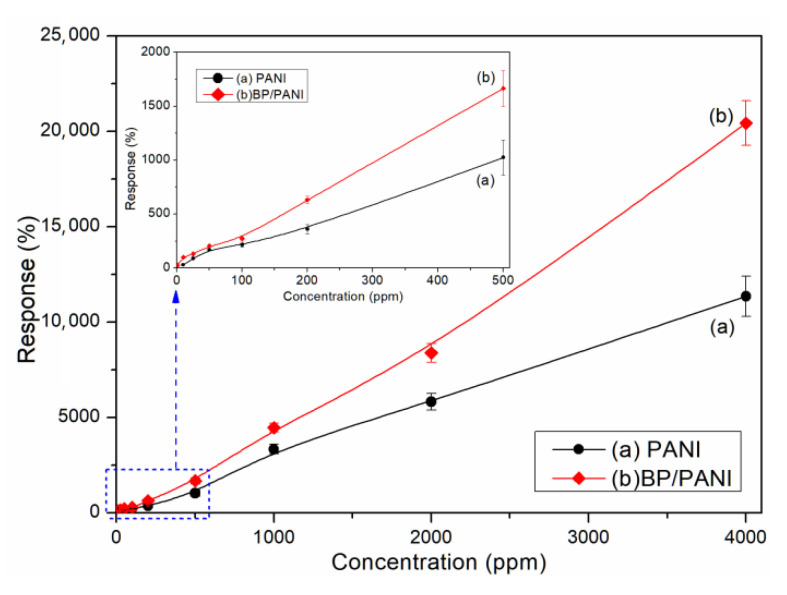
Response values of (a) PANI and (b) BP/PANI thin film sensors exposed to different concentrations of NH_3_ at room temperature.

The NH_3_ response (S) of the sensor can be defined as follows [9]:

(2) $$S=\frac{\Delta R}{R_{air}}=\frac{R_{G}-R_{air}}{R_{air}}\times 100\%$$

where *R_G_* and *R_air_* are the resistances at selected NH_3_ concentrations and in air. The sensor achieved 90% of the total resistance change, which is defined as the response and recovery time, respectively. As shown in Figure 5, the response curves of PANI and BP/PANI sensors are derived from Figure 4a,b. It can be clearly seen that the values of PANI and BP/PANI show near linearity for NH_3_ over a wide range from 1 to 4000 ppm, and the inset graph shows a range from 1 to 100 ppm. The experimental results show that the response of the BP/PANI thin film is clearly higher than that of the PANI thin film. Furthermore, the low detection limits of BP/PANI and PANI thin film are 1 ppm and 10 ppm for NH_3_ gas, respectively. In other words, the BP added to PANI could enhance the sensitivity and enlarge the detection range for the sensing of NH_3_ gas compared to that of PANI as a key role.

**Figure 6:**
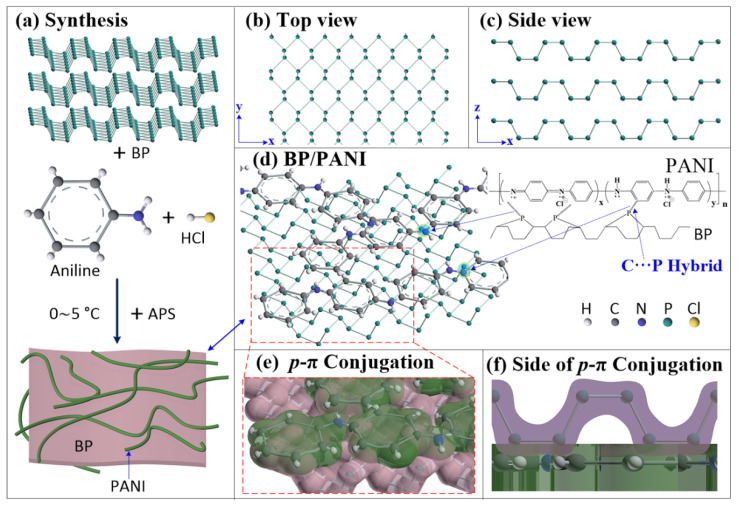
Schematic illustration for (**a**–**c**) atomic structure of BP, (**a**) polymerization of PANI or BP/PANI, (**d**) C⋯P hybrid of BP/PANI, and (**e**,**f**) *p-π* conjugation system (this is exaggerated for illustration purposes).

Until now, there has been no unified conclusion able to identify enhanced sensitivity and an enlarged detection range for NH_3_ gas sensing. Possible explanations for this are mainly listed as follows. Firstly, as aforementioned, BP has been added into aniline monomers solution to form the BP/PANI nanocomposite, resulting in BP/PANI thin films that have higher specific surface area than that of PANI. Therefore, the sensitivity and detection range of BP/PANI are notably enhanced by the gas diffusion because of its higher exposure area and penetration depth for NH_3_ molecules [9], which was also confirmed by the SEM study completed earlier. Secondly, the crystal structure of BP is a strongly folded honeycomb sheet with “troughs” running along the y axis [19], as shown in Figure 6a–c. This is a very special structure that is beneficial to enhance the diffusion of NH_3_ molecules into BP/PANI thin films, along the troughs. Thirdly, there is a synergetic effect [10] and/or *p–π* conjugation system in the BP/PANI nanocomposite through the C⋯P hybrid [26], as shown in Figure 6d. This is to say, the electron clouds of BP and PANI may overlap, as shown in Figure 6e,f. Thus, the electron cloud area of BP/PANI for charge interaction between NH_3_ molecules and *p*/*π* electrons is higher than that of PANI. Finally, the BP added to PANI may form a *p*–*p* hole accumulation/depletion isotype heterojunction at the PB/PANI interface, which can enhance the sensing response for NH_3_ [42,43,44]. On the one hand, the Fermi level of BP and PANI are 3.9 eV [45] and 2.8–3.28 eV [42,43], respectively. A new equilibrium of the Fermi level is achieved due to carriers transferring from each other when the PANI is coated onto BP. In the depletion layer, the protons of BP/PANI are captured by NH_3_ molecules, which reduces the doping level of PANI. In this case, the depletion region of BP/PANI is widened, i.e., the total resistance of BP/PANI is increasing with gas concentration. On the other hand, according to the relative position of work functions [43], the holes are gradually accumulated on the PANI side due to the lower work function (4.4 eV) and are correspondingly depleted on *p*-type BP side with higher work function (4.56–5.16 eV) [46]. In this case, when the sensor is exposed to NH_3,_ it firstly reacts with the surface PANI nanofibers, and the dedoping (deprotonation) process is attributed to the capture of protons from PANI to NH_3_ molecules, increasing the total resistance and sensitivity of BP/PANI.

### 3.3. Response, Recovery, and Selectivity

**Figure 7:**
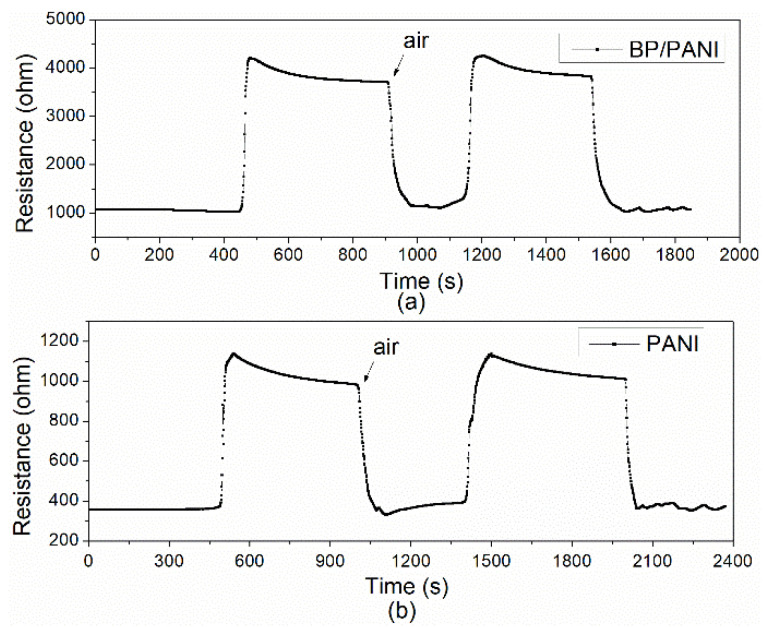
Typical response and recovery curves of (**a**) PANI and (**b**) BP/PANI.

It is well known that the response–recovery time is an important parameter for gas sensors. The response and recovery transients of the PANI and the BP/PANI nanocomposite were tested under pure air and 100 ppm NH_3,_ at 25 °C in a rotation. The response and recovery transients of the PANI and BP/PANI sensors are shown in Figure 7. The test results indicate that the response time and recovery time of BP/PANI are ca. 28 s and 46 s, respectively. Simultaneously, the response time and recovery time of PANI are ca. 41 s and 36 s, respectively. Additionally, the resistance changes of PANI and BP/PANI sensors were ca. 66% and ca. 76%, respectively. Thus, it is clearly seen that all sensors show good reproducibility and fast response–recovery behavior for NH_3_ sensing.

**Figure 8:**
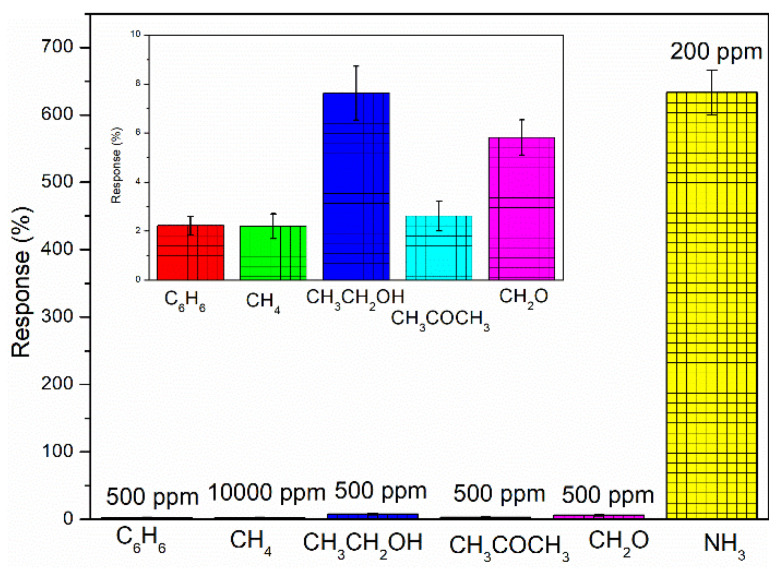
The selectivity of BP/PANI thin film to various gases.

Selectivity is also an important parameter for the gas sensors. It can be seen from Figure 8 that the selectivity of the BP/PANI sensor versus different gases was tested at room temperature. Several common gases in daily living and from production, such as benzene (C_6_H_6_), methane (CH_4_), ethanol (CH_3_CH_2_OH), acetone (CH_3_COCH_3_), and formaldehyde (CH_2_O), were tested to evaluate the cross-sensitivity effect. The experimental results presented that all of the gases had no significant cross-sensitivity effect on BP/PANI NH_3_ sensing. Thereby, the BP/PANI thin film showed excellent NH_3_ selectivity in comparison with other gases.

## 4. Conclusions

In summary, a BP/PANI nanocomposite was synthesized using in situ chemical oxidative polymerization of aniline monomers with BP, which was then fabricated into IDTs through the drop-coating method and used as sensitive thin films for NH_3_ detection. Next, the electrical properties of the BP/PANI thin films were studied in a large detection range, from 1 to 4000 ppm, including conduction mechanism, response, reproducibility, and selectivity. The test results indicated that the BP/PANI thin film sensor showed higher sensitivity and a larger detection range than that of PANI. The sensitivity and detection range of BP/PANI were notably enhanced due to the BP, which plays an important role in the gas diffusion. The BP added to PANI that may enlarge the specific surface area, obtained a special trough structure for gas channels, and formed a *p–π* conjugation system and *p*–*p* heterojunctions, which are beneficial to increase the response of BP/PANI to NH_3_ sensing. Meanwhile, in order to support the above discussion, the structure and morphology of the BP/PANI were characterized using FTIR, UV−vis, TEM, and SEM, respectively. The sensor showed good reproducibility and a fast response and recovery behavior for NH_3_ sensing in this work. This route may offer the advantages of the sensor having a simple structure, low cost, easy assembly, and operation at room temperature. However, over the past few decades, the long-term stability of PANI has been an unsolved problem in the field and in research. Further research on exploring the stability of BP/PANI nanocomposites will be summarized in elsewhere.

## Data Availability

The data presented in this study are available on request from the first author.
